# Social Interactions of Juvenile Brown Boobies at Sea as Observed with Animal-Borne Video Cameras

**DOI:** 10.1371/journal.pone.0019602

**Published:** 2011-05-04

**Authors:** Ken Yoda, Miku Murakoshi, Kota Tsutsui, Hiroyoshi Kohno

**Affiliations:** 1 Graduate School of Environmental Studies, Nagoya University, Furo-cho, Chikusa-ku, Nagoya, Japan; 2 Graduate School of Marine Science and Technology, Tokai University, Shimizu, Shizuoka, Japan; 3 Institute of Oceanic Research and Development, Tokai University, Shimizu, Shizuoka, Japan; 4 Okinawa Regional Research Center, Tokai University, Uehara, Taketomi, Okinawa, Japan; Cajal Institute, Consejo Superior de Investigaciones Científicas, Spain

## Abstract

While social interactions play a crucial role on the development of young individuals, those of highly mobile juvenile birds in inaccessible environments are difficult to observe. In this study, we deployed miniaturised video recorders on juvenile brown boobies *Sula leucogaster*, which had been hand-fed beginning a few days after hatching, to examine how social interactions between tagged juveniles and other birds affected their flight and foraging behaviour. Juveniles flew longer with congeners, especially with adult birds, than solitarily. In addition, approximately 40% of foraging occurred close to aggregations of congeners and other species. Young seabirds voluntarily followed other birds, which may directly enhance their foraging success and improve foraging and flying skills during their developmental stage, or both.

## Introduction

In many animals, social interactions play a crucial role in the growth of young individuals, and studies have shown that naïve animals or young individuals change their behaviour in the presence of others [1]. These social interactions and the associated social learning are expected to be adaptive, allowing individuals to acquire pertinent information by exploiting the experience and knowledge of others, without the trial-and-error costs associated with nonsocial learning [2]. As early development exerts direct effects on subsequent growth and fitness [3], examining how young individuals respond to others in the wild could be important. In principle, naïve or young individuals should have a propensity to approach conspecifics and/or other species to gain social information inadvertently provided by others [4]. In particular, they would follow or join a group of knowledgeable individuals to increase their own foraging [5] or migration success [6]. However, although social interaction is observable under controlled conditions in the laboratory and sometimes in the wild, observing social interactions of highly mobile animals in inaccessible environments is difficult.

Recently, developments in animal-borne still cameras [7] and video recorders [8] have begun to provide “organism-eye” views of animals. These devices can record social interactions of animals living at sea (e.g. group foraging of penguins, [9]). In particular, video recorders are promising device for the research in social interactions related to young individuals, as still cameras lose important behavioural details such as quick feeding actions [10]. However, the size of the video recorder strictly limited its application to volant seabirds (e.g. [11]). In addition, as juvenile seabirds slowly grow during developmental stages that last for weeks or months (e.g. altricial birds) [12], they may show age-related social behaviour during periods of growth. In this regard, as small video recorder has the short lifespan due to small battery size [8], it is difficult to cover the age-related change of behaviour over time.

In this study, we deployed miniaturised video recorders on juvenile brown boobies (*Sula leucogaster*) that were hand-reared beginning as chicks to examine how social interactions between tagged juveniles and other birds affected their flight and foraging behaviour during the post-fledging dependence period (PFDP; 1–3 months). The reared boobies made round trips between the sea and nest where they begged for food from researchers during the PFDP [12], [13], therefore, we could easily deploy and recover video recorders on them for the trips at sea.

## Materials and Methods

### Ethics statement

Our study was conducted under the approval of the Nature Conservation Division, Okinawa, Japan.

### Methods

**Figure 1:**
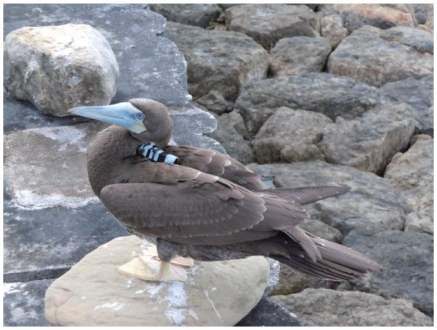
Juvenile brown booby fitted with a video recorder. The recorder was attached to the back of the booby to provide bird's eye view of the environment. The overall weight of the device was less than 2.5% of the mass of the birds. These hand-raised boobies made round-trips between sea and nest during the post-fledging dependence period.

This study was carried out in 2010 on Nakanokamishima Island (24°11′N, 123°34′E) and Okinawa Regional Research Centre (ORRC), Tokai University, Iriomote Island (24°19′N, 123°41′E), Japan. Nakanokamishima Island hosts brown boobies, brown noddies (*Anous stolidus*), streaked shearwaters (*Calonectris leucomelas*) and three other species of seabirds. For more detailed information on our hand-raising methods, see our earlier paper [13]. We brought three chicks of unknown sexes to the ORRC (4–15 days old). All of the birds recognised us as parents, and we raised them and fed tropical fish to them when they begged. After fledging, small plastic base was attached on the back feathers with adhesive tape (Tesa, Hamburg, Germany) and glue (Loctite 401). A data logger was attached using cable ties which enter through holes of a recorder and beneath the feathers glued with the base. As such, video recorder can be repeatedly removable by cutting the cable ties during the study period (Fig. 1). The video lens faces forward to provide bird's eye view of the environment. We used a video camera data logger (LY30, 19×68 mm, Benco, Taiwan) after improving its waterproof sealing. This camera had a 280 mAh Li-polymer battery and 4 GB memory and could record for 2 h. The resolution was 736×480 pixels, with a frame rate of 30 frames per second. The overall weight was 27 g, which was less than 2.5% of the mass of the birds. The birds made trips out to sea during the day and returned to the nest at dusk. We deployed the data loggers in early morning and recovered them at night by cutting the cable ties; we then downloaded the movie data to computers. The birds did not appear to be negatively affected by the video recorder and the frequent handling by researchers.

In addition, we recorded the trip duration of the juveniles to calculate the proportion of time recordable on our cameras. We defined flight duration between the time when the bird took flight from and the time of landing on water or land. We defined flight as flying 15 s or more in the air. We defined tagged birds as engaged in “chasing” flight when the camera recorded tagged birds flying with other birds. We defined solitary flight as flight other than chasing flights. We used breast plumage to distinguish between adults and juveniles. We identified objects filmed before and after 5 s from the time a tagged bird plunged into water and distinguished the objects into brown boobies, other seabird species, physical objects, and fish. We defined social foraging as plunging in the presence of two or more animals.

The incidence of chasing flights was analysed in relation to the days since fledging using a generalised linear mixed model (GLMM) with a logit link and binomial error distribution. In addition, we designed a linear mixed model (LMM) of flight duration, treating flight type (chasing/solitary) and days since fledging as fixed factors. We also designed a LMM treating the bird that was chased (adult/juvenile) and days since fledging as fixed factors. We regressed the incidence of social foraging using a GLM with a logit link and binomial error distribution that treated the days since fledging as a fixed factor. For all models, we treated the individual bird as a random effect.

Data were analysed using R version 2.7.2 [14]. LMMs and GLMMs were run using the lme4 package [15]. The significance of LMM fixed effects was obtained from 100,000 Markov chain Monte Carlo (MCMC) simulations, performed using the pvals.fnc function in the languageR package [16]. The significance of the fixed effects was obtained from the *z* value of the GLMMs.

## Results

**Figure 2:**
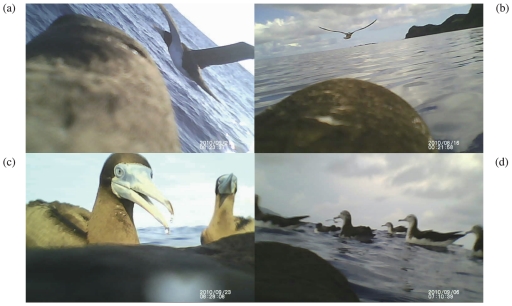
Images obtained from miniaturised video cameras attached to the backs of juvenile brown boobies. A: A bird flying with an adult booby. The bird's head is at the bottom of the camera's field of view, B: a bird flying with another tagged juvenile, C: a bird resting on water surface with other brown boobies, D: in the flock of streaked shearwaters.

On average, birds fledged 94 days after hatching and left the nest 95 days after fledging. The video data logger was attached 18 days on average to each bird during the PFDP. The trip duration of the three birds was 3.2±2.9 h (*n* = 256). Thus, our cameras could cover more than 60% of trip duration during the PFDP. The videos showed social activities of tagged boobies that flew with other birds (see electronic supplementary material, movie S1), were resting with other species on the sea surface (Fig. 2), and plunged into the sea in areas where other birds were resting.

**Figure 3:**
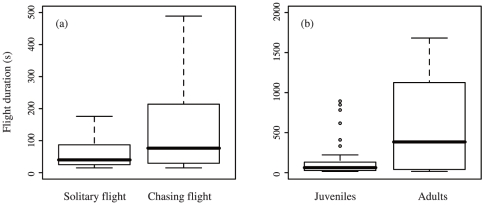
Flight duration of juvenile brown boobies with their congeners. A: solitary flights and chasing flights, B: chasing flights of other juveniles and adult brown boobies. In (A), chasing flights include flights with birds of unknown age. Outliers were omitted from the Figure (A) for a clearer display.

The incidence of chasing flight did not change with days after fledging (table S1, electronic supplementary material). Flight duration increased significantly with the number of days after fledging (table S1). In addition, the flight duration of tagged juveniles was longer when they were chasing other birds (206±303 s, *n* = 72) than in solitary flights (102±205 s, *n* = 221; Fig. 3A, table S1). Tagged birds also flew for a longer period with adults (605±682 s, *n* = 6) than with other juveniles (138±202 s, *n* = 55; Fig. 3B, table S1). Plunge dives (*n* = 489) occurred in the presence of other brown boobies (25.8%), other seabird species (10.8%), floating objects (12.1%) and fish (1.6%). Other species consisted of brown noddies (55%), streaked shearwaters (3.8%), both streaked shearwaters and brown noddies (3.8%), both brown noddies and black-naped tern *Sterna sumatrana* (1.9%) and unidentified species (35.8%). The incidence of plunge dives in the presence of brown boobies did not change with days after fledging, whereas those in the presence of other species increased as juveniles approached independence (table S1).

## Discussion

Our study showed that juveniles did not increase the frequency of chasing flights with age during the PFDP, but did increase flight duration in the presence of others as they approached independence. This indicates that they gradually acquired flight skills [12] and could follow other individuals that they encountered at sea. Also, juveniles flew longer when they followed adults rather than juveniles. In general, adults are more knowledgeable foragers [17]; thus, juveniles may benefit directly by following adults and learning the location of food. Additionally, by following better foragers, juveniles may refine their own foraging skills through practice. We could not determine whether juveniles flew longer just because they followed good flyers, or whether they distinguished knowledgeable adults from juveniles and preferentially followed adults to better prey patches.

About 40% of the plunge diving of the tagged juveniles occurred close to congeners and other species, mainly brown noddies. Seabirds can locate prey locations by observing the foraging behaviour of other individuals. This local enhancement strategy [18] is especially important for poorer foragers, i.e. juveniles. As plunging boobies are an attractive signal to several seabird species [19], juvenile brown boobies can detect foraging conspecifics easily. In addition to conspecifics, other species can also provide prey information to boobies. In fact, brown boobies and brown noddies have some overlap in prey [20] that might be driven to the surface by large predators like tuna [21]. Interestingly, the incidence of plunge diving in the presence of other species increased with the age of the juveniles. The local enhancement signals of brown noddies may be weaker due to their inconspicuous feeding method (i.e. surface dipping or prey snatching) and/or their foraging range may be larger than that of brown boobies [22], [23]. Therefore, juvenile brown boobies can encounter brown noddies during late-stage PFDP, as the boobies gradually acquire a larger home range size (HK & KY, unpublished data).

Our study showed that juvenile brown boobies followed conspecifics and other species possibly to gain public information on foraging grounds. However, the use of social information is also an essential help for every forager, not only for juveniles [4]. Therefore, to examine whether following other birds is a specific to the age class of juvenile brown boobies, we need to deploy our video system on several age classes, including adult boobies, and compare the properties of social interactions between them.

In conclusion, we revealed that fledglings changed their behaviour at sea in the presence of other birds by deploying video recorders on free ranging seabirds for the first time. Young seabirds follow other birds voluntarily, which may enhance their foraging success directly or result in improved foraging and flying skills during the developmental stage or both.

## Supporting Information

Table S1 **Summary of derived model terms from GLMs and LMMs for probability of chasing flights, flight duration during chasing flights, chasing flight duration with adults or juveniles and probability of plunge diving in the presence of brown boobies and other species.** Provided are estimates of the coefficient and its standard error (*b* ± *s.e*), as well as level of significance (*P*).
(XLS)

Movie S1 **Movie from video cameras attached to juvenile brown boobies** ***Sula leucogaster*** **.** Two scenes are presented from cameras on different birds: chasing a juvenile and joining flocks of other species (brown noddies *Anous stolidus* and streaked shearwaters *Calonectris leucomelas*) at a feeding site. The video camera was attached to the back of the booby. Hence, the bird's head sometimes appears at the bottom of the camera's field of view. Large camera shake occurs during flapping flight, whereas intermittent gliding produces a relatively small shake. The resolution of the movie was downsized due to the server capacity.
(MOV)
